# High incidence of diagnosis with syphilis co-infection among men who have sex with men in an HIV cohort in Ontario, Canada

**DOI:** 10.1186/s12879-015-1098-2

**Published:** 2015-08-20

**Authors:** Ann N. Burchell, Vanessa G. Allen, Sandra L. Gardner, Veronika Moravan, Darrell H. S. Tan, Ramandip Grewal, Janet Raboud, Ahmed M. Bayoumi, Rupert Kaul, Tony Mazzulli, Frank McGee, Sean B. Rourke, on behalf of the OHTN Cohort Study Team

**Affiliations:** Ontario HIV Treatment Network, Suite 600, 1300 Yonge Street, Toronto, ON M4T 1X3 Canada; Dalla Lana School of Public Health, University of Toronto, Toronto, Canada; Public Health Laboratories, Public Health Ontario, Toronto, Canada; Department of Medicine, University of Toronto, Toronto, Canada; Toronto General Research Institute, University Health Network, Toronto, Canada; Division of Infectious Diseases, St. Michael’s Hospital, Toronto, Canada; Institute of Health Policy, Management and Evaluation, University of Toronto, Toronto, Canada; Division of General Internal Medicine, St. Michael’s Hospital, Toronto, Canada; Centre for Research on Inner City Health, Li KaShing Knowledge Institute, St. Michael’s Hospital, Toronto, Canada; Mount Sinai Hospital, Toronto, Canada; AIDS Bureau, Ontario Ministry of Health and Long Term Care, Toronto, Canada; Department of Psychiatry, University of Toronto, Toronto, Canada; Li Ka Shing Knowledge Institute, St. Michael’s Hospital, Toronto, Canada; Department of Family and Community Medicine, St. Michael’s Hospital, Toronto, Canada

## Abstract

**Background:**

The re-emergence of syphilis among HIV-positive gay and other men who have sex with men (MSM) requires vigilance. We estimated incidence of and risk factors for first and subsequent syphilis diagnoses among MSM in HIV care in Ontario, Canada.

**Methods:**

We analyzed data from 2,280 MSM under follow-up from 2006 to 2010 in the Ontario HIV Treatment Network Cohort Study (OCS), a multi-site clinical cohort. We obtained syphilis serology results via record linkage with the provincial public health laboratory. Rates were calculated using Poisson regression.

**Results:**

First syphilis diagnoses occurred at a rate of 2.0 per 100 person-years (95 % CI 1.7, 2.4; 121 cases) whereas the re-diagnosis rate was 7.5 per 100 person-years (95 % CI 6.3, 8.8; 136 cases). We observed higher rates over time and among men who were aged <30 years, receiving care in the two largest urban centers, or had a previous syphilis diagnosis. Syphilis diagnosis was less common among Indigenous men, men with higher CD4 cell counts, and, for first diagnoses only, among men with less than high school education.

**Conclusions:**

Compared to reported cases in the general male population, incidence of a new syphilis diagnosis was over 300 times greater among HIV-positive MSM but year-to-year changes reflected provincial trends. Re-diagnosis was common, suggesting treatment failure or re-infection. Novel syphilis control efforts are needed among HIV-positive MSM.

## Background

After years of decline, many urban centres have reported concerning rises in case reports of infectious syphilis in North America, Europe, Australia, and Asia [1–5]. Untreated, infections enter an asymptomatic latent stage that can last for many years; potential complications include tissue destruction of any organ [6] including neurosyphilis, a potentially life threatening complication [7]. Local epidemiology suggests that these outbreaks are largely occurring among gay and other men who have sex with men (MSM). In Ontario, Canada, reports of infectious syphilis rose from 0.4 to 5.9 cases per 100,000 from 2001 to 2012, with virtually all (96 %) cases among males [8]. Most male cases (88 %) reported sex with other men and HIV co-infection was noted in 40 % of cases [1]. In contrast, HIV prevalence is 23 % and 11 % among MSM in Toronto and Ottawa, respectively [9, 10], suggesting over-representation of HIV-positive men in syphilis transmission networks. Internationally, higher rates of bacterial STI infection have been noted among HIV-positive compared to HIV-negative MSM [11–13]. Nevertheless, we are unaware of published reports of syphilis incidence in HIV-positive MSM populations in our setting or elsewhere in Canada.

Investigation of syphilis incidence requires consideration of re-infection to fully characterize the patterns of population-level transmission and to design interventions. New syphilis cases among HIV co-infected men commonly have a past history of previous syphilis infection [8, 14, 15]. Epidemiological characterization of the rate of first versus subsequent syphilis infections could help to determine to what extent syphilis epidemics are concentrated in core groups experiencing repeated infections.

Our primary objectives were to estimate the incidence of first and subsequent syphilis diagnoses in a large cohort of HIV-positive MSM and to identify risk factors for these diagnoses. As we had full access to syphilis test results but were challenged by incomplete recording of syphilis staging information in medical charts, a secondary objective was to evaluate the use of laboratory-based definitions for their potential in monitoring rates of new syphilis diagnoses.

## Methods

We analyzed data from the Ontario HIV Treatment Network Cohort Study (OCS). The province of Ontario has publicly-funded, universal access to medically-necessary health care services. An estimated 32,547 Ontarians have been infected with HIV since reporting began in 1985 up to the end of 2011; 77 % were men who reported sex with men as their potential route of HIV acquisition [16]. We have previously described the study design [17]. Briefly, the source population consisted of people aged 16 and older diagnosed with HIV infection and who received specialty HIV care at one of 10 hospital- or community-based practices. Approximately 43 % of clinic patients were active participants (range 20–79 % across clinics) [17]. Participants provided written consent. Clinical data recorded as part of participants’ routine health care were abstracted from medical records. Beginning in 2007, participants were interviewed annually using structured questionnaires. The study protocol and instruments received ethical approval from the University of Toronto Human Subjects Review Committee and from the individual study sites.

### Viral load and syphilis testing

We obtained laboratory data for HIV viral load and syphilis through record linkage with the Public Health Ontario Laboratories, the sole provider of such testing in Ontario. Serology is the primary diagnostic methodology used for syphilis testing. Since 2006, the testing algorithm includes screening with a treponemal test, the Abbott chemiluminescent immunoassay (CMIA), followed by confirmatory testing with the RPR, TPPA, and FTA-ABS tests [18]. To ensure comparability of results across years, we excluded person-time for years prior to 2006.

### Incidence definitions

We calculated the annual incidence density of syphilis diagnoses. We use the term “diagnosis” rather than “infection” because syphilis tests were ordered based on clinical decisions rather than standardized testing protocols and intervals between tests varied, both of which would tend to underestimate true infections. A similar approach was used in the province of British Columbia, Canada [15]. Incident cases were defined using syphilis serologic results only. We previously reported that medical chart data were insufficient for staging among the majority (57 %) of patients whose serology suggests active infection [19].

Men who had a negative serology result in 2006–2010 were eligible for inclusion in the calculation of incidence of a first diagnosis of syphilis. The denominator was person time beginning at the first negative test or year immediately following HIV diagnosis, whichever was later. The numerator was the number of men with subsequent reactive syphilis serology.

Men with a reactive syphilis serology test were considered at risk for re-diagnosis at the first non-acute rapid plasma reagin (RPR) test (≤1:8) in 2006–2010, or the year immediately following HIV diagnosis, whichever was later. Men diagnosed with syphilis during follow-up were considered at risk for re-diagnosis beginning 120 days after the previous event, under the assumption that all would have received adequate treatment by such time given clinical guidelines [6] and average frequencies of HIV care in our cohort. In sensitivity analyses, we varied the timing of re-entry to the risk set at 90, 120, 150 or 180 days after the previous diagnosis. These intervals were more stringent than previous research that considered cases at risk of a new diagnosis within 42 days [15]. Our primary definition was a four-fold rise in RPR titre. In sensitivity analyses, we used a more conservative definition requiring a four-fold rise in titre to 1:16 or greater.

Left- and right-censoring was applied to incidence calculations. For men who were diagnosed with HIV in 2006 or later, we left-censored and excluded person-time in years up to and including the year of HIV diagnosis because we wished to exclude syphilis detected prior to or concomitant with HIV diagnosis. Men who were lost-to-follow-up were right-censored as of their last known clinic visit and men known to have died were censored at the date of death; all others were censored on December 31, 2010.

### Analysis

We analyzed data available as of December 2011, at which time 5933 participants had enrolled. We restricted the analysis to participants under observation at any time from 2006 to 2010 (1,686 excluded), for whom there was successful record linkage to the HIV viral load database at the PHOL (260 excluded), and who were men who either self-identified as gay or bisexual or reported sex with men as an HIV risk factor (*n* = 2,775 men). The year 2011 was excluded to maximize availability of clinical data as manual chart reviews were incomplete for that year. We further restricted the sample to men who had at least one syphilis test in 2006–2010 (429 excluded), who were diagnosed with HIV prior to 2010 (13 excluded), and who contributed person-time to either the calculation of first or re-diagnosis (62 excluded). The final sample size was 2,271 MSM. We conducted all statistical analyses using SAS version 9.3 (SAS Institute, Inc., Cary, North Carolina). *P*-values were two-sided. Statistical significance was determined using the conventional *p*-value of < 0.05.

We first used descriptive statistics to characterize men included in the analyses of first and re-diagnosis with syphilis according to their age, year of HIV diagnosis, race/ethnicity, education, income, region of Ontario, clinical and antiretroviral treatment status.

Next we calculated and compared annualized incidence densities using Poisson regression and time-updated covariates in PROC GENMOD. To account for recurrent events, we used a generalized estimating equations (GEE) framework with an auto-regressive correlation structure. This approach adjusts standard errors for correlation among repeated observations within individuals. We modeled the total number of events using an offset for the number of person-years of follow-up time. We used intercept-only models to estimate crude rates with 95 % confidence intervals. We conducted separate analyses for first and re-diagnoses (including a sensitivity analysis using our more conservative definition of re-diagnosis), as well as a combined analysis of the incidence of syphilis diagnosis, for which first and subsequent events were counted equally.

Rates were compared using incidence rate ratios (RR) calculated by exponentiating the parameter estimates. We analyzed calendar time as a categorical and continuous variable to verify whether the association was linear. Race/ethnicity, education and income were analyzed as time-invariant variables. Age, region, viral load, CD4 cell count, and antiretroviral treatment status were time-updated each calendar year. All considered covariates were included in the final model; observations with missing data on covariates were excluded. To evaluate evidence for differences in risk factors for first or subsequent syphilis diagnosis, we tested the statistical significance of an interaction term between the risk factor of interest with an indicator variable for past syphilis diagnosis.

To further address our secondary objective to evaluate the use of laboratory-based definitions for their potential in monitoring rates of new syphilis diagnoses, we wished to compare our estimated rates in the cohort to population-based rates with specific attention to temporal trends. The ideal comparison would be syphilis rates among the entire population of MSM in Ontario but such data were unavailable. Therefore, we compared our observed rates to those that were available via public health surveillance, namely population-based rates of reported infectious syphilis among males in Ontario [20].

## Results

At baseline, the 2,271 men were aged 46 years, on average (Table 1). The majority self-identified as gay, were of white race/ethnicity, had been diagnosed with HIV prior to 2006, and received HIV care in Toronto, the largest city in the province. Most (83.8 %) had initiated antiretroviral treatment. Nearly two-thirds had undetectable HIV viral load and the mean CD4 cell count was 497 cells/mm^3^. Participants were followed a median of 2.5 years for a sum total of 7,275 person-years. Men included in the analysis of syphilis re-diagnosis were largely similar to men included in the analysis of first diagnosis, with some exceptions (Table 1). Among men with documented past syphilis who were at risk for re-diagnosis, a greater proportion self-identified as gay and received care in Toronto; they were also slightly older at baseline (mean 47.1 versus 45.2 years, *P* <0.0001).

**Table 1:** Characteristics of HIV-positive MSM included in the analysis, OHTN Cohort Study, 2006–2010

	Analysis of first syphilis diagnosis		Analysis of syphilis re-diagnosis		Combined analysis of new syphilis diagnosis	
	n	%	n	%	n	%
Total N	1799	100	586	100	2271	100
Mean age at baseline (SD) (a)	45.2	9.2	47.1	10.5	45.9	9.5
Sexual orientation						
Gay	1360	75.6	493	84.1	1752	77.1
Bisexual	129	7.2	27	4.6	151	6.6
Heterosexual	75	4.2	7	1.2	81	3.6
Unknown (b)	235	13.1	59	10.1	287	12.6
Race						
White	1311	72.9	423	72.2	1654	72.8
Black/African	54	3	27	4.6	77	3.4
Multiple	138	7.7	52	8.9	177	7.8
Indigenous	111	6.2	20	3.4	126	5.5
Other	116	6.4	51	8.7	157	6.9
Unknown	69	3.8	13	2.2	80	3.5
Education (c)						
High school or less	395	22	111	18.9	493	21.7
Trade school or college	498	27.7	179	30.5	633	27.9
University	680	37.8	242	41.3	871	38.4
Unknown (b)	226	12.6	54	9.2	274	12.1
Annual personal income (c)						
Less than $20,000 CDN	565	31.4	188	32.1	716	31.5
$20,000 to $59,999 CDN	631	35.1	213	36.3	799	35.2
$60,000 CDN or more	353	19.6	122	20.8	452	19.9
Unknown (b)	250	13.9	63	10.8	304	13.4
Region where receiving HIV care						
Toronto	1224	68	474	80.9	1598	70.4
Ottawa	193	10.7	50	8.5	237	10.4
Elsewhere in Ontario	382	21.2	62	10.6	436	19.2
Year of HIV diagnosis						
Median (IQR)	1997	(91,03)	1997	(91,03)	1996	(91,03)
Diagnosed in 2006–09	177	9.8	56	9.6	214	9.4
Clinical status at baseline (a)						
Initiated antiretroviral treatment	1952	86.0	1546	85.9	491	83.8
Mean CD4 cell count/mm^3^ (SD)	497.1	261.6	491.3	252.4	497.1	261.6
Undetectable viral load (d)	1173	65.2	363	61.9	1473	64.9

*MSM* Men who have sex with men, *SD* Standard deviation, *IQR* Interquartile range

(a) Baseline was the later of January 1, 2006 or the date of enrolment in the cohort

(b) Unknowns include men who were not interviewed

(c) As reported at the participant’s first interview in 2007–2010, available for 1967/2271 men

(d) Undetectable viral load defined as <50 copies/mL in 2006–2009 and <40 copies/mL in 2010

### First syphilis diagnosis

**Fig. 1:**
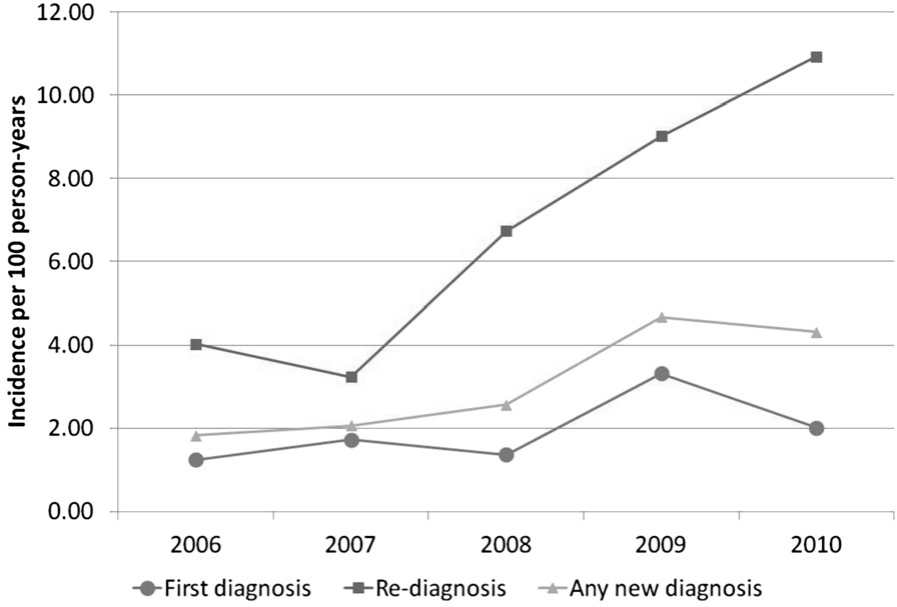
Rate of new syphilis diagnoses among HIV-positive MSM, OHTN Cohort Study, 2006–2010. Re-diagnosis defined as a four-fold rise in RPR titre. See text for details

Among the 1,799 men with initially negative serology, first syphilis diagnoses occurred at a rate of 2.0 per 100 person-years (95 % CI 1.7, 2.4; 121 cases). Incidence increased on average 17 % per calendar year with the highest rate observed in 2009 (Fig. 1; Table 2). We observed statistical evidence of rate differences according to age, region, and education, such that the highest rates were among young men under the age of 30, men receiving care in Toronto, and men with more than high school education (Table 3).

**Table 2:** Rates of new syphilis among HIV-positive MSM in the OCS compared to males in Ontario

	HIV-positive MSM, OHTN Cohort Study (OCS)				Ontario males (a)	
Year	Rate of first diagnosis per 100PY (95 % CI)	Rate of re-diagnosis per 100PY (95 % CI) (b)	Rate of any new diagnosis per 100PY (95 % CI)	Percent change from preceding year	Rate of new infectious syphilis case per 100PY	Percent change from preceding year
2006	1.2 (0.6, 2.5)	4.0 (1.9, 8.4)	1.8 (1.1, 3.0)	---	0.0055	---
2007	1.7 (1.1, 2.7)	3.2 (1.8, 5.8)	2.1 (1.5, 2.9)	13.2	0.0061	10.9
2008	1.4 (0.9, 2.1)	6.7 (4.6, 9.8)	2.6 (1.9, 3.4)	24.3	0.0065	6.6
2009	3.3 (2.5, 4.4)	9.0 (6.6, 12.3)	4.7 (3.8, 5.8)	82.8	0.0107	64.6
2010	2.0 (1.4, 2.9)	10.9 (8.3, 14.4)	4.3 (3.5, 5.4)	−7.9	0.0112	4.7

*PY* Person-years, *CI* Confidence interval

(a) Population-based rates of reported infectious syphilis among males in Ontario, Canada. Source: [20]

(b) Re-diagnosis defined as an observable four-fold rise in RPR titre. See text for details

**Table 3:** Risk factors for a new diagnosis of syphilis among HIV-positive MSM, OHTN Cohort Study, 2006–2010

	First syphilis diagnosis		Re-diagnosis of syphilis (a)		Any new syphilis diagnosis	
Risk factor	Rate per 100PY (95 % CI)	Adjusted RR (95 % CI)	Rate per 100PY (95 % CI)	Adjusted RR (95 % CI)	Rate per 100PY (95 % CI)	Adjusted RR (95 % CI)
Time (1-year increase)		1.3 (1.1, 1.5)		1.4 (1.2, 1.6)		1.3 (1.1, 1.4)
Age						
<30 years	6.8 (4.2, 11.1)	4.5 (1.9, 10.3)	18.7 (10.9, 32.3)	3.7 (1.9, 7.2)	9.5 (6.6, 13.7)	3.0 (1.7, 5.4)
30–49 years	2.2 (1.8, 2.7)	1.7 (1.0, 2.9)	9.8 (8.0, 12.0)	2.4 (1.6, 3.7)	3.7 (3.2, 4.3)	1.6 (1.1, 2.3)
50+ years	1.1 (0.7, 1.7)	1.0	3.8 (2.7, 5.4)	1.0	1.9 (1.5, 2.5)	1.0
Race						
White	1.9 (1.6, 2.4)	1.0	7.4 (6.1, 9.0)	1.0	3.2 (2.8, 3.7)	1.0
Black	2.3 (0.8, 6.0)	0.9 (0.3, 2.6)	7.8 (3.5, 17.3)	0.8 (0.4, 1.8)	3.9 (2.1, 7.3)	1.0 (0.5, 1.8)
Indigenous	0.8 (0.3, 2.5)	0.2 (0.02, 1.3)	1.9 (0.3, 13.6)	0.4 (0.1, 2.8)	0.9 (0.4, 2.5)	0.25 (0.06, 0.97)
Other/unknown	2.8 (1.9, 4.0)	1.1 (0.7, 1.8)	8.3 (5.8, 11.9)	0.9 (0.5, 1.4)	4.2 (3.2, 5.4)	0.9 (0.6, 1.3)
Education						
High school or less	0.9 (0.5, 1.6)	0.4 (0.2, 0.8)	5.7 (3.7, 8.8)	0.8 (0.4, 1.4)	1.9 (1.4, 2.7)	(b)
Trade school or college	2.7 (2.0, 3.6)	1.0 (0.6, 1.6)	8.9 (6.8, 11.8)	1.1 (0.7, 1.7)	4.2 (3.5, 5.2)	
University	2.4 (1.8, 3.1)	1.0	7.5 (5.8, 9.7)	1.0	3.6 (3.0, 4.4)	
Annual personal income						
Less than $20,000 CDN	1.9 (1.4, 2.7)	1.1 (0.6, 2.0)	7.7 (5.8, 10.3)	0.8 (0.5, 1.4)	3.3 (2.7, 4.1)	1.2 (0.8, 1.8)
$20,000–$59,999 CDN	2.3 (1.7, 3.0)	1.1 (0.7, 1.9)	6.7 (5.0, 8.9)	0.6 (0.4, 1.0)	3.3 (2.7, 4.1)	0.9 (0.6, 1.4)
$60,000 CDN or more	1.9 (1.3, 2.9)	1.0	9.4 (6.8, 12.9)	1.0	3.7 (2.9, 4.8)	1.0
Region receiving HIV care						
Toronto	2.6 (2.2, 3.1)	4.0 (1.6, 10.3)	8.4 (7.0, 10.0)	2.9 (0.9, 9.0)	4.1 (3.6, 4.7)	4.7 (2.1, 10.6)
Ottawa	1.1 (0.5, 2.6)	0.7, (0.1, 6.4)	3.1 (1.2, 8.3)	1.2 (0.3, 5.2)	1.5 (0.8, 2.9)	2.2 (0.7, 7.0)
Elsewhere in Ontario	0.4 (0.2, 1.0)	1.0	3.1 (1.4, 6.9)	1.0	0.8 (0.4, 1.4)	1.0
Maximum viral load in year						
Detectable	2.9 (2.2, 3.7)	1.3 (0.8, 2.3)	8.8 (6.9, 11.1)	1.0 (0.6, 1.5)	4.4 (3.7, 5.3)	1.1 (0.8, 1.6)
Undetectable	1.6 (1.2, 2.1)	1.0	7.0 (5.5, 8.8)	1.0	2.8 (2.3, 3.3)	1.0
Minimum CD4 in calendar year						
<350 cells/mm^3^	2.1 (1.5, 2.9)		8.4 (6.4, 11.1)		3.7 (3.0, 4.5)	
350–499 cells/mm^3^	2.6 (1.9, 3.5)		9.0 (6.7, 12.2)		4.1 (3.3, 5.1)	
500 cells/mm^3^ or greater	1.5 (1.0, 2.1)		6.4 (4.7, 8.7)		2.6 (2.0, 3.2)	
Per 100 unit increase		0.93 (0.85, 1.01)		0.93 (0.85,1.0)		0.92 (0.86, 0.99)
On antiretroviral treatment						
Yes	1.7 (1.4, 2.4)	0.9 (0.5, 1.7)	7.0 (5.8, 8.5)	0.8 (0.5, 1.4)	2.9 (2.5, 3.4)	0.9 (0.6, 1.3)
No	3.8 (2.7, 5.4)	1.0	9.6 (6.6, 13.7)	1.0	5.3 (4.1, 6.8)	1.0
Past syphilis diagnosis						
Yes					19.7 (14.8, 26.2)	3.5 (2.2, 5.8)
No					2.8 (2.4, 3.2)	1.0

*PY* Person years, *CI* Confidence interval, *RR* Rate ratio calculated using Poisson regression adjusted for all variables shown

(a) Re-diagnosis defined as a four-fold rise in RPR titre. See text for details

(b) In the analysis of any new syphilis diagnosis, there was a statistically significant interaction between education and history of past syphilis diagnosis (*P* = 0.04). Adjusted RRs (95 % CI) for education level *among men with no past syphilis diagnosis* were 0.42 (0.25, 0.73) for high school or less and 0.88 (0.62, 1.3) for trade school/college compared to men with university. *Among men with a past syphilis diagnosis*, the adjusted RRs were 1.4 (0.69, 8.4) for high school or less and 1.3 (0.71, 2.3) for trade school/college compared to men with university

### Re-diagnosis of syphilis

Among the 586 men with reactive serology indicative of past syphilis infection, there were 136 case events among 114 men using our primary definition of re-diagnosis, i.e., a four-fold rise in RPR titre. Among men with a re-diagnosis, most (82 %) had one, 16 % had two, and 2 % had three re-diagnoses of syphilis. Incidence of re-diagnosis was 7.5 per 100 person-years (95 % CI 6.3, 8.8) if all 136 cases were considered re-diagnoses. Eighty-four percent of case events (114/136) met our more conservative definition of re-diagnosis of a four-fold rise in titre to at least 1:16; on average, the titre rose to 1:64 (IQR 1:16 – 1:128). As expected, incidence was somewhat lower at 6.2 per 100 person-years (95 % CI 5.2, 7.5) when using the conservative re-diagnosis definition. Case counts for re-diagnosis were virtually unchanged with varying time of re-entry to the risk set (range 134–137 cases for our primary re-diagnosis definition; range 112–115 cases for re-diagnosis using the more conservative definition). Incidence of re-diagnosis increased an average of 36 % per calendar year with the highest rate observed in 2010 (Fig. 1; Table 3).

As for rates of first diagnosis, re-diagnosis rates were higher among younger men and men receiving care in Toronto (Table 3). However, there was no difference according to education. Rate ratios were largely unchanged when we used our more conservative definition of re-diagnosis (data not shown).

### Combined analysis of first and subsequent syphilis diagnoses

Overall, incidence of a new syphilis diagnosis (first documented diagnosis or subsequent diagnosis) was 3.3 per 100 person-years (95 % CI 2.9, 3.7; 257 cases) with a trend for increasing incidence over time (Fig. 1; Table 3). Men with a history of previous syphilis were at greater risk of a new syphilis diagnosis compared to men with no such history (adjusted RR = 3.5, 95 % CI 2.2, 5.8). There was evidence of modest within-subject correlation (rho = 0.12 in an intercept-only model for observations 1 year apart). Among cases, 22 % were among men who had previously had syphilis.

Risk factors for any new syphilis diagnosis were consistent with those observed in separate analyses of first and re-diagnoses. Although Indigenous men had the lowest rates of first and re-diagnoses, it was only in the combined analysis that the difference had adequate precision to reject the null hypothesis (adjusted RR = 0.25, 95 % CI 0.06, 0.97). Similarly, the relation between increasing CD4 cell count and lower incidence of syphilis diagnosis was sufficiently precise to rule out chance association only in the combined outcome model. Education was the sole risk factor showing statistical evidence of effect modification. Compared to men with university education, men with high school education or less were less likely to have a new diagnosis of syphilis if they had no previous history of syphilis (adjusted RR = 0.42, 95 % CI 0.25, 0.73); however, the rate ratio was null among men with a past history of syphilis (adjusted RR = 1.4, 95 % CI 0.69, 8.4; *P*-value for interaction = 0.04).

### HIV viral load among cases

We characterized HIV viral load patterns among all 257 new syphilis cases. For the test on or immediately preceding the date of syphilis diagnosis, 59 % had undetectable viral load, 8 % had detectable but low-level viremia (<1,000 copies/mL), 29 % had a viral load between 1,000 and 100,000 copies/mL, and 4 % had a viral load >100,000 copies/mL. Among cases with detectable viral load, 52 % (65/126) had been taking antiretroviral medications according to their medical chart records.

## Discussion

Among gay and other MSM in HIV care in Ontario, Canada, the rate of new syphilis diagnoses rose from 1.8 per 100 person-years in 2006 to 4.3 per 100 person-years in 2010. These rates are over 300 times greater than those reported for the general male population in Ontario over the same period [1], but are consistent with those observed internationally among HIV-positive MSM, including in the United States [21], Australia [12] and Europe [11, 22]. Importantly, the rates we documented in the cohort are under-estimates since they were measured in routine practice rather than by active screening programs.

Strengths of our analysis include a large sample of HIV-positive men with known sexual orientation and record linkage with syphilis serology results from the sole laboratory that conducts such testing for the province. Although a high proportion of reported syphilis cases in our setting have been identified as HIV-positive MSM [8], our findings advance knowledge by calculating rates of new syphilis diagnosis—first and re-diagnoses—using a known denominator that was well characterized according to sociodemographic, HIV risk, and clinical features.

Potential limitations include under-ascertainment of true syphilis infection due a lack of standardized testing practice and selection bias due to volunteer participation. Only 55 % of MSM in the cohort undergo syphilis testing at least once a year [19]. We may have inaccurately classified “first” syphilis diagnoses because as many as 25 % of cases may serorevert if treated successfully in early infection [6]. Nevertheless, the overall rate of new syphilis diagnoses would not have been affected by false classification of re-diagnoses as “first” diagnoses since both were counted equally. Cohort participants are generally representative of HIV diagnoses in Ontario in terms of sex, geographic region, age at diagnosis, and HIV exposure category [16]; however, they under-represent the recently diagnosed and include only those successfully linked to HIV care. Compared to non-OCS clinic patients, participants tend to be older, were diagnosed in the more distant past, and are generally healthier as measured by CD4 cell count and viral load [23]. Altogether, we believe that the true value for the rate of syphilis infection (rather than diagnosis) among all HIV-positive MSM in Ontario was higher than what we observed.

Regional and temporal patterns in the cohort mimicked provincial findings in the general male population from notifiable disease surveillance [1, 8]. The year-to-year rate changes we observed were consistent with those reported overall in Ontario. Moreover, rates in the cohort were highest among men living in Toronto and Ottawa, as expected according to provincial case reports [1, 8]. (It should be noted, however, that syphilis diagnosis rates were non-negligible outside of these cities at 0.8 per 100 person-years (95 % CI 0.4, 1.4)). Our estimates were relatively robust to varying definitions of re-diagnosis. In sum, these consistencies suggest that the laboratory-based case definitions we used may be adequate for monitoring trends in syphilis co-infection among HIV-positive MSM and for determining whether public health interventions produce rate decreases in this population.

Men with HIV who experience a syphilis diagnosis may require additional supports to prevent re-infection and greater vigilance to diagnose it according to our findings and those of others. First diagnosis occurred at a rate of 2.7 per 100 person-years (95 % CI 1.7, 2.4) whereas re-diagnoses occurred at a rate of 4.8 per 100 person-years (95 % CI 3.7, 5.5), such that men with a previous documented history of syphilis infection were 3.5 times more likely to have a new diagnosis according to our multivariable model. Treatment of syphilis results in loss of protective immunity and resusceptibility to infection [24]. Although we cannot entirely rule out treatment failure, we believe that the majority of re-diagnoses were re-infections given our case definitions and standards of care for syphilis treatment for HIV patients [6]. Among syphilis case reports, history of previous infection was more common among HIV-positive than HIV-negative individuals in British Columbia and in Ontario [8, 14, 15], and a similarly high rate of re-diagnosis, at 2 per 100 person-years, was documented in British Columbia [15]. Future research should explore underlying causes of heightened risk for re-infection. Hypothesized explanations include ongoing risk behaviour, perhaps due to beliefs that syphilis is a minor health problem (thus little concern regarding re-infection). Nevertheless, in our cohort, 78 % of new syphilis diagnoses were among men without laboratory evidence of past syphilis infection, suggesting only moderate concentration of ongoing risk among men with past infection.

New syphilis diagnoses were most common among young men at a rate of 9.5 per 100 person-years (95 % CI 6.6, 13.7) for those under the age of 30. Declining risk with age is a common feature of STI epidemiology among HIV-positive [7, 25–27] and general populations [1, 2]. Targeting syphilis control interventions only to young men, however, may miss a substantial number of new cases. Although diagnoses were three times less likely among men over the age of 50, they still occurred at a rate of 1.9 per 100 person-years (95 % CI 1.5, 2.5).

We observed few racial or ethnic differences in syphilis, with the exception that Indigenous men were *less likely* to be diagnosed with syphilis. This is in contrast to findings from the United States where African American men tend to have higher rates of syphilis diagnosis compared to White men [2, 25, 26]. Our sample sizes were small for race/ethnicity categories other than White; confirmation with larger samples would be prudent. Nor did we detect dramatic differences in syphilis diagnosis rates according to income. Among men with no past history of syphilis, men with university education were more likely to have a first diagnosis of syphilis compared to men with high school or less; however, there were no educational differences in risk for syphilis re-diagnosis. Similarly, a spatial epidemiologic analysis of syphilis-HIV co-infection case rates in Toronto observed no significant rate differences according to neighborhood-level measures of residential instability, material deprivation, dependency, or ethnic concentration [14].

After adjustment for sociodemographic characteristics, syphilis diagnosis rates were equivalent among men who had or had not initiated antiretroviral treatment and who did or did not have undetectable viral load. Nevertheless, men with high CD4 cell counts had lower syphilis diagnosis rates; this could be a marker for better overall health, differences in sexual behaviours and networks, or alternatively may be explained by reverse causation, as CD4 decline has been noted following syphilis acquisition [28–30].

The acquisition of syphilis among HIV-positive MSM in our setting suggests that sexual behaviours are occurring that could allow for HIV transmission to partners. In recent years, the proportion of men on antiretroviral treatment and with suppressed viral load has greatly increased in our cohort [31], and preliminary findings suggest that many syphilis acquisitions occur during encounters between HIV-positive men (“poz” sex) [32]; both phenomena would mitigate the potential risk of onward HIV transmission. Nevertheless, syphilis co-infection may promote secondary HIV transmission because HIV infectiousness can be enhanced at this time [6, 33–35]. HIV viral load is a primary predictor of infectiousness [36] and it may rise during syphilis co-infection [28, 29], although there is limited evidence that seminal viral load rises among men successfully treated with modern antiretroviral regimens [37, 38]. Among syphilis cases in our cohort, one third had plasma viremia of 1,000 copies/mL or higher at the time of syphilis diagnosis; half of such cases were among men taking antiretroviral medications. Our group will pursue explanations for these findings in a future paper, notably whether syphilis co-infection preceded a rise in viremia, whether ART had been initiated too recently to have achieved an undetectable VL, or whether men had suboptimal medication adherence around the time of syphilis infection and diagnosis.

## Conclusions

Our findings have implications for ongoing efforts to improve gay men’s sexual health. We recommend more concerted efforts to monitor rates of new syphilis infections among MSM in Ontario, as such data have been absent in our setting. The observance of high rates of syphilis among men with HIV in our cohort may help to mobilize resources to promote timely testing and treatment, thereby mitigating syphilis sequelae, health care costs, and onward spread to sexual partners. If syphilis is detected during its early stages (<1 year), treatment is an inexpensive single injection of benzathine penicillin G [6, 39]. Patterns of syphilis diagnoses in our cohort and among reported cases in Toronto [14] suggest that the syphilis epidemic is mature and not restricted to a core region or sociodemographic group. The co-epidemics of HIV and syphilis are complex and will likely require multiple, broad-scale approaches that promote behaviour change, partner notification, and frequent testing that ensures rapid diagnosis and treatment, thereby preventing onward transmission [40–42].
